# Examining corruption risks in the procurement and distribution of COVID-19 vaccines in select states in Nigeria

**DOI:** 10.1186/s40545-023-00649-7

**Published:** 2023-11-13

**Authors:** Obinna Onwujekwe, Charles Orjiakor, Pamela Ogbozor, Ifunanya Agu, Prince Agwu, Tom Wright, Dina Balabanova, Jillian Kohler

**Affiliations:** 1 Leslie Dan Faculty of Pharmacy, University of Toronto, Toronto, Canada; 2 Health Policy Research Group, Department of Pharmacology and Therapeutics, College of Medicine, University of Nigeria, Enugu, Nigeria; 3 Department of Health Administration and Management, University of Nigeria, Enugu, Nigeria; 4 Department of Psychology, University of Nigeria, Nsukka, Nigeria; 5 Department of Psychology, University of Toronto, Scarborough, Canada; 6 Department of Psychology, Enugu State University of Science and Technology, Enugu, Nigeria; 7 Department of Social Work, University of Nigeria, Nsukka, Nsukka, Nigeria; 8 Department of Global Health and Development, London School of Hygiene and Tropical Medicine, London, UK; 9 Transparency International Global Health Programme, Transparency International, London, UK

**Keywords:** Accountability, Coronavirus, Corruption, COVAX, COVID-19, Transparency, Vaccines

## Abstract

**Background:**

Public health emergencies raise significant concerns about corruption and accountability; however, these concerns can manifest in different ways across diverse locations. For instance, more developed countries with a stronger rule of law may experience more corruption in vaccine procurement, whereas developing countries may experience more corruption at the point of distribution and delivery to end users. This research focuses on corruption concerns in Nigeria, specifically examining the procurement and distribution of COVID-19 vaccines.

**Methods:**

This paper utilizes a scoping review and a qualitative research approach. Key informants (*n* = 40) involved in the procurement and distribution of COVID-19 vaccines across two states in Nigeria were interviewed. Findings from the scoping review were summarized, and collected data were inductively coded and analysed in themes, revealing clear examples of implementation irregularities and corruption in the country’s COVID-19 vaccination processes.

**Results:**

Vaccination programme budgeting processes were unclear, and payment irregularities were frequently observed, resulting in vaccinators soliciting informal payments while in the field. Recruitment and engagement of vaccination personnel was opaque, while target vaccination rates incentivized data falsification during periods of vaccine hesitancy. Accountability mechanisms, such as health worker supervision, vaccination data review, and additional technical support provided by donors were implemented but not effective at preventing corruption among frontline workers.

**Conclusions:**

Future accountability measures should be evidence-driven based on findings from this research. Personnel recruitment, contracting, budgeting, and remuneration should focus on transparency and accountability.

## Introduction

Corruption is “the misuse of entrusted power for private gain” and, more broadly, the misuse of office privileges and positions to undermine and cheat people and systems in ways that divert institutions from their core goals of service, accountability, public trust, and confidence [1]. In October 2020, United Nations Secretary-General Antonio Guterres declared that corruption was one of the greatest risks to making progress in the COVID-19 pandemic [2]. Governments globally aimed to contain the devastating effects of COVID-19 through rapidly evolving measures, including movement restrictions, closure of domestic and international borders, physical distancing rules, lockdowns, mandatory use of facial coverings, and prioritization of public health efforts.

The use of vaccines was a major countermeasure in the fight against COVID-19. Many COVID-19 vaccines were developed, procured by governments, and deployed around the globe. Vaccine development and distribution were seen by many as prerequisites to lifting emergency restrictions, and citizens supported their governments rapidly securing vaccines using non-standard procedures. The desperation of citizens to exit restrictions and live “normal” lives provided opportunities for government actors to exploit emergency procurement processes, which favoured rapid procurement to curb the spread of the virus over greater checks and balances to prevent corruption [3].

Procurement and distribution of health products such as vaccines are highly susceptible to corruption, especially during public health emergencies when more emphasis is placed on availability of medical supplies than due diligence [4]. There is also evidence that more money comes with more corruption, which is why allocations to the top spending priorities in the health system—human resource management and procurement—tend to be more prone to corruption than other areas [5].

**Fig. 1:**
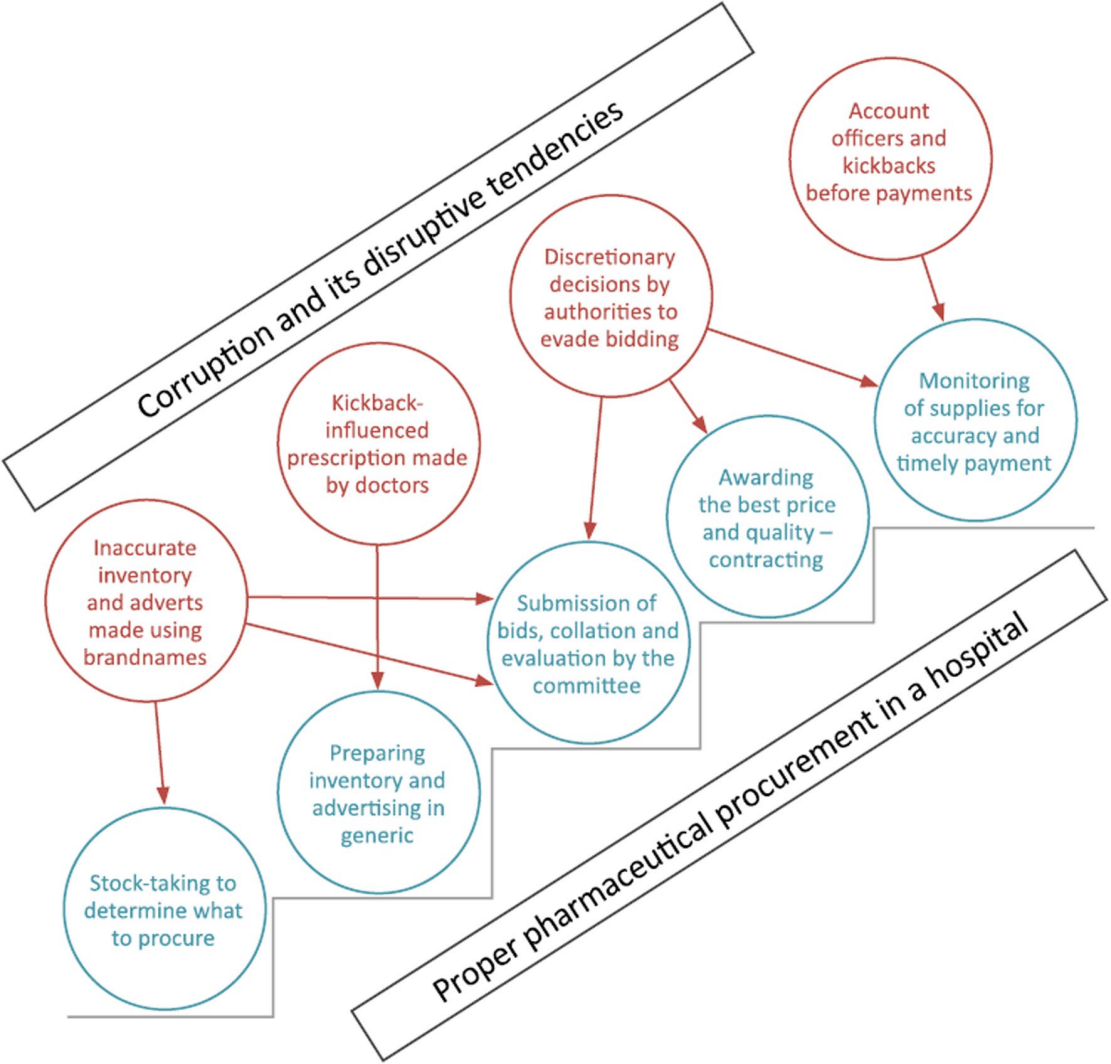
Corruption in standard pharmaceutical procurement services compared to proper pharmaceutical procurement [5]

The literature in this field has documented diverse types of procurement corruption [5]. For instance, corrupt activities during the bidding phase of procurement may include inaccurately estimating the demand for a particular product or service, circumventing procedures guiding bidding, and deliberately tailoring tender documents to favour a particular bidder based on social connections or being compromised by bribes. In other stages of procurement, corruption could involve delivering different supplies from what was approved, making discretionary decisions about what is to be purchased and which supplier is to be engaged, or demanding bribes before paying suppliers. Figure 1 compares a typical corrupt procurement system to a non-corrupt one. Based on early findings of this research, some of these issues were evident in COVID-19 vaccine procurement [6].

In Nigeria, COVID-19 vaccine procurement has faced allegations of corruption and unaccountability [2]. Beyond procurement, corruption has been documented in the administrative provision and implementation of essential services that require proof of vaccination [4, 7], with vaccine-hesitant persons who needed to travel and use essential services acquiring falsified vaccination records [8, 9]. Other suspected forms of vaccine-related corruption include the theft and diversion of vaccines to informal markets and solicitation of informal payments for vaccines [10].

There is a paucity of information on the accountability issues that arise as COVID-19 vaccines reach their end users. Studying vaccine distribution can help inform accountability processes and help ensure that vaccines are allocated and delivered in a fair, efficient, and equitable manner based on the principles of human rights and public health. Understanding accountability issues associated with vaccine distribution can help prevent discrimination, corruption, and waste that may undermine the effectiveness and legitimacy of vaccination efforts. Such understanding can also help identify and address the gaps and challenges that may arise in the complex and dynamic process of vaccine distribution, such as logistical issues, supply chain disruptions, regulatory barriers, and ethical dilemmas.

Corruption in vaccine distribution can have serious negative consequences for public health, fairness, trust, and financial resources. Several analyses have weighed and highlighted the risks of corruption in the procurement and distribution of COVID-19 vaccines [2, 10]; however, many of these studies were theoretical and speculative. This paper provides new information on the corruption risks surrounding COVID-19 vaccine procurement and distribution in Nigeria, focusing on accountability, corruption, and transparency at the state and local government levels. Nigeria received its first batch of COVID-19 vaccine in early March 2021 from COVAX, a global collaboration involving among others, governments, multinational health agencies, scientists, manufacturers, that constituted the vaccine pillar of the Access to COVID-19 Tools (ACT) Accelerator initiative. The ACT Accelerator was tasked with driving the development, production, and equitable access to COVID-19 tests, treatments, and vaccines globally.

After this, Nigeria began a vaccination campaign to inoculate its citizens [11]. Amid the global and local concerns of vaccine insufficiency, especially for low-resource regions, this study was designed to investigate the accessibility of COVID-19 vaccines in this low-resource region. This study also highlights accountability and anti-corruption mechanisms that local stakeholders utilized to minimize corruption risks, as well as factors or conditions that enabled or constrained these mechanisms.

## Methods

### Study area

The study was conducted in two states in the southern region of Nigeria between January and March 2023. The states have remained anonymous to protect the confidentiality of key informants. State A has an estimated population of about 3 million as of 2017, and State B has an estimated population of about 4.4 million as of 2019 (Table 1). We report non-exact estimates for the details of each state to ensure confidentiality.

**Table 1:** Geographic and health facility data of State A and State B

	State A	State B	Source
Population	over 4.3 million	over 3 million	National Bureau of Statistics
Land area	over 7,000 km^2^	over 5,000 km^2^	National Bureau of Statistics
Local Government Areas (LGAs)	over 15	over 10	National Bureau of Statistics
Number of health facilities	over 800	over 500	Ministry of Health, State B State
Number of primary health centres	over 510	over 500	Ministry of Health, State B State

The states of interest were selected based on their proximity to the authors based in Nigeria and their COVID-19 case load. The selected states had one of the highest cases of COVID-19 cases and the other state had one of the lowest number of residents vaccinated as at the time of data collection [12, 13]. The difference in COVID-19 case load was relevant for state selection, as evidence indicates that a region’s case load may be associated with its vulnerability to corruption [10, 14].

### Study design and participants

Data were collected through a scoping literature review and qualitative interviews with key stakeholders in the two selected states.

### Scoping literature review

A scoping review of the literature was conducted to identify published and grey literature that highlighted anti-corruption, transparency, and accountability (ACTA) issues in COVID-19 vaccine procurement and distribution. Documents were included in the review if they identified and discussed ACTA issues or if they implemented or proposed remedies to ACTA problems related to COVID-19 vaccine procurement and distribution. Documents were excluded if they discussed COVID-19 and its global impact generally without implicitly or explicitly identifying any ACTA issues of concern. The literature captured in the review was published between 2020 and 2022.

#### Information sources and literature search strategy

A team of researchers independently generated key concepts for the literature search. Key concepts included accountability, transparency, corruption, and anti-corruption. The names of COVID-19 vaccines used anywhere in the globe were also included among the key concepts. We allowed a global outlook but honed the concepts to highlight concerns for low-income regions, especially the sub-Saharan region of Africa. Nigeria remained a key focus of the search. From the pool of key concepts, Boolean operators were generated and revised several times to optimize search outcomes. Researchers covered nine databases (Embase, PubMed, ResearchGate, Medline, Scopus, Web of Science, Yahoo, Google, and Google Scholar). Five researchers used the finalized Boolean operators to search for documents of interest, including academic articles, online news articles, technical reports, and local policy documents that featured COVID-19 vaccine accountability concerns. Researchers also reviewed citations of interest in the identified literature to retrieve additional relevant documents.

#### Selection process

For three weeks, the five researchers independently searched the databases using an identical set of Boolean operators. All researchers conducted a preliminary screen of each identified document’s abstract and executive summary, followed by a full-text screen to identify documents that discussed issues of interest for inclusion. The researchers then pooled all retrieved articles and removed duplicates.

#### Data collection process

A review template was developed to analyse key parts of the procurement process: financing and budgeting, procurement strategy/solicitation/award of contract, contract implementation, distribution and delivery of vaccine, and monitoring and evaluation. Complete details of the information source (e.g., bibliographic materials) were documented. Researchers independently completed data extraction templates for each included document before pooling the results, removing duplicates, and adding details that independent reviewers may have omitted. A written narrative summarizing findings was then synthesized from the data extraction master list based on author discussions and consensus.

### In-depth interviews

An in-depth interview (IDI) guide was used for interviews with different categories of stakeholders to elicit responses on their perspectives on, involvement with, and inefficiencies experienced in COVID-19 vaccine procurement and distribution in States A and B. Study participants were purposively drawn from the following categories based on their experiences and involvement in COVID-19 vaccine deployment in the selected states: (i) state-level participants from the State Ministry of Health (SMOH) and State Primary Health Care Development Agency (SPHCDA); (ii) Local Government Area (LGA)-level participants including senior officials in the LGA health department; (iii) health care facility-level participants who were directly involved in vaccine administration, and (iv) service users consisting of community members who received a COVID-19 vaccine.

The research team collectively developed the IDI guides and piloted them in another state using a similar study population. Findings from the pilot study were useful in revising the tool before the main study. Interviews were scheduled with respondents and conducted at locations selected by the respondents to ensure that they could communicate freely. All interviews were audio recorded. Most were conducted in English, and a few in the local Indigenous language of State A and State B. Interviews conducted in the local language were translated to English, and all interviews were transcribed verbatim. The entire data collection and reporting process was aligned to the Consolidated Criteria for Reporting for Qualitative Research (COREQ) [15].

### Data collection

The State Primary Health Care Development Agency (SPHCDA) of the two states provided research support in helping identify the actors from the state and local governments who played active roles in the COVID-19 vaccine distribution process. A directory that included the names and contact information of those to be interviewed was formulated based on this information. We adhered strictly to the list, which contained “information-rich” people and those with authority on the subject.

### Data analysis

Data analysis was guided by a phenomenological approach focused on eliciting the lived experiences of the respondents alongside their expert insights [16]. All responses were audio recorded and transcribed. Researchers reviewed and analysed transcripts based on codes that were generated inductively and informed by our research questions. Following review and agreements among the researchers, the following final coding themes were established: (i) corrupt practices in COVID-19 vaccine deployment; (ii) efforts by local establishments to control corruption in COVID-19 vaccine deployment; and (iii) constraints to ACTA mechanisms in COVID-19 vaccine deployment.

## Results

### Scoping review of literature

#### International factors that may foster corruption in COVID-19 vaccine access

The studies included in our review spanned between 2020 and 2022, reflecting the years of the COVID-19 pandemic. The research team retrieved and reviewed a total of 40 documents. The review found that the urgent need to procure COVID-19 vaccines provided opportunities for corruption throughout the development and supply chain. Conflicts of interest arising from governments working closely with vaccine manufacturers may have impaired objective review in downstream vaccine selection and funding processes [2]. The resulting alignment between governments and the pharmaceutical industry meant that agreements on terms of engagement and contract details were inaccessible and confidential, creating information asymmetries between officials involved in vaccine procurement decision-making and the public [17, 18]. This limited information meant that the public was less aware of government commitments of public funds, and civil society groups were less aware of who to hold accountable and less able to identify when decision-makers failed to meet their commitments [18]. Governments and the pharmaceutical industry justified this information secrecy by claiming it is needed to protect proprietary rights.

Human Rights Watch observed that the high demand for and low supply of COVID-19 vaccines, facilitated by constricted supply chains for required raw materials, enabled high-income countries to maintain a stranglehold on vaccine supplies by pre-ordering doses that severely limited low-income countries’ access to COVID-19 vaccines [19]. High-income countries and countries with vaccine manufacturing capacity also adjusted their export policies to ensure that their citizens had access to vaccines before allowing vaccines to be exported [20]. The scarcity created by the inequitable distribution of COVID-19 vaccines was identified as a driver of vaccine theft and the emergence of fake or substandard vaccine products [10], as well as financial leaks, nepotism, and favouritism in the procurement system [2].

COVID-19-related misappropriations and procurement scandals were reported to have resulted in the loss of millions of dollars in both high-income countries, such as the United Kingdom, and low- and middle-income countries, such as Kenya and South Africa [20, 21]. These misappropriations were often traced to high-level officials and were associated with poor population-wide access to vaccine-related resources. One commentary identified that discriminatory resource distribution practices across sectors (including education, agriculture, and health) typical of African settings were also observed in the distribution of COVID-19 vaccines and resources [22]. Cronyism and tribalism may similarly influence vaccine allocation and distribution. A perception of acute vaccine shortages led to vaccine corruption scandals, the development of black and opaque markets for vaccines, and bribes to allow certain individuals to jump vaccination queues [23].

Poor testing capacity and surveillance can lead officials to underestimate the cases of COVID-19 and underprepare for its impact, thus preventing actors from procuring vaccines when they are most needed [24]. More demand pressure may then build up, increasing incentives to engage in corrupt practices to access limited supplies. Decision-making related to allocating vaccines to priority groups is particularly vulnerable to corruption risks, including conflicts of interest, nepotism, and favouritism [2]. Health care professionals exercising their discretion to dispense legitimate products based on nepotism and favouritism may further contribute to product scarcity and increase opportunities for substandard or falsified vaccines to proliferate [2].

#### Factors that may foster corruption in COVID-19 vaccine access in Nigeria

A need for rapid access to COVID-19 vaccines meant that procurement and bidding best practices were often overlooked [25]. The Nigeria Vaccine Policy, released in September 2021, demonstrates how procurement speed was prioritized over process accountability [26].

Within Nigeria, vaccine procurement concerns were complicated by events related to the COVID-19 pandemic that made people suspicious of the government. Food and other relief materials meant to alleviate the burden of the pandemic were spotted in warehouses across the country, undistributed [27]. The public perceived that these resources were stockpiled for politicians and political parties, causing massive protests and revolts about the government pandemic response and plummeting trust in the government’s COVID-19 policies. In the midst of these events, many Nigerians avoided COVID-19 vaccines and instead attempted to unlawfully acquire vaccination cards [28]. This was particularly prevalent among people seeking to travel abroad, as vaccine certificates were often required for international travel [28]. Health officials were implicated in vaccine certificate falsification, with rogue officials charging high informal fees to issue vaccination certificates to individuals who had not yet been vaccinated, yet health managers reported that they were unable to gather sufficient evidence to arrest corrupt officials [28].

Weak leadership and decision-making within the Nigerian health sector, as well as existing accountability issues, meant that infrastructure to store and distribute vaccines was frequently unavailable [5]. Decision-makers were also unable to recommend COVID-19 vaccines that were suitable for the level of infrastructure available in the Nigerian system [27]. Even after doses were procured, slow vaccine distribution increased public distrust and fostered suspicions that doses were being reserved for the elite class [29]. For example, a state governor’s wife was reported to travel abroad to get a COVID-19 vaccine shot from the United States while Nigeria was struggling to access COVID-19 vaccines [30]. Distrust also arose when international staff of multinational aid agencies, including those connected to COVAX, appeared to have earlier access to vaccines than local staff [31].

#### Factors that improve accountability and transparency in COVID-19 vaccine procurement and distribution

Several researchers and policy analysts have suggested mechanisms to bolster vaccine procurement and distribution. Although many of these mechanisms have not been evaluated for effect or impact, they nonetheless reflect standard ACTA practices and are expected to have positive effects. Implementing corruption risk assessments into routine government monitoring processes was proposed to identify potential entry points for corruption [2, 32]. Since pharmaceutical procurement and distribution, especially during emergencies, present opportunities to make money, privilege seekers deploy deviant mechanisms to secure supply/service deals and make undue profits, risk assessments can increase public awareness of corruption risks and help countries prevent it.

Digital platforms that regulate and track vaccine importation and distribution, as well as share quality surveillance information across regulatory agencies, were also identified as tools to increase monitoring of vaccine supply chains, thus enhancing procurement-related ACTA measures [32].

#### Factors that may hinder accountability and transparency in COVID-19 vaccine procurement and distribution

Opacity around COVID-19 vaccine procurement decision-making resulted in limited public access to relevant information and data sharing, which hindered transparency in vaccine procurement and distribution [18, 19, 32]. This was the case both locally and internationally, with scholars lamenting the lack of public information to track who should be held accountable for COVID-19 vaccine inefficiencies [31]. Similarly, using special commissions to negotiate the purchase of COVID-19 vaccines, as opposed to usual procurement mechanisms, also hindered transparency and increased the risk of corruption in the distribution of pandemic-related funds [2].

The complexity of the COVAX governance structure, as well as its shortfalls in delivering vaccines as projected, was also considered a serious accountability problem. COVAX held lofty ideals of efficient and equitable vaccine distribution, but it has been criticized for enabling vaccine nationalism and failing to distribute vaccines to disadvantaged populations within poorer countries [17]. Researchers point to COVAX’s relatively weak governance mechanisms, which allowed wealthier countries and powerful private pharmaceutical companies to wield influence and secure resources to their advantage [17, 33, 34].

#### Mechanisms that can help reduce the risk of corruption in the COVID-19 vaccine procurement and distribution

Several recommendations to improve ACTA in COVID-19 vaccine distribution were identified in the literature. Many of the suggestions were not exclusive to COVAX, but were designed to improve efficiency of COVID-19 vaccine procurement and distribution more broadly.

One paper suggested waiving patent rights on vaccine technology and making it more freely available, thereby boosting vaccine production and availability, and lessening the scarcity that creates a climate that encourages corruption [20]. While this proposal would infringe on the intellectual property rights of manufacturers, Amnesty International suggests that expanding and diversifying manufacturing through sharing intellectual property and open, non-exclusive licensing are important for a more accountable and transparent vaccine procurement and distribution system [35].

Other papers also strongly recommended strengthening vaccine procurement and distribution oversight and monitoring, including using domestic activists, civil society groups, or anti-corruption agencies to demand accountability from government [2, 20, 36, 37]. Since countries typically have local activists that push for health rights, some proposals suggested leveraging these activists to demand accountability from the actors involved in vaccine procurement and distribution. Others underscored the role of establishing accountability and reporting mechanisms to monitor financial disbursement processes and verify receipt of appropriate funds, as well as diversifying monitoring teams to include balanced gender proportions [2]. Specialized committees with a robust anti-corruption mandate to oversee and disburse emergency funds, distribute and prioritize vaccines, and monitor vaccine-related programmes were also recommended [2, 10]. Donor support of ACTA mechanisms to ensure the proper distribution of vaccines in recipient countries was highlighted as particularly important [38]. When distributing vaccines to vulnerable groups, monitoring distribution channels to ensure that supplies would not be diverted or tampered with was recommended [10].

Normal health procurement procedures that are employed during non-health emergencies should not be jettisoned and replaced with entirely new emergency committees. Centralized procurement procedures were considered effective in securing affordable pricing and reducing the public purchasing of substandard vaccines [32]. Routine procurement best practices, such as harmonizing packaging using a global standard (including a centralized track-and-trace system), can reduce administrative and financial burdens associated with repackaging products at the national level. Digital platforms that enhance within and between country communication about resource needs (e.g., by publishing information about procurement coordination, surveillance, operational support and logistics, timelines, and prioritization of different groups), such as the District Health Information Software 2 (DHIS2), can further strengthen ACTA in procurement [32]. Regional networks and systems that promote timely data sharing can also contribute to improving procurement efficiency [32, 39]. ACTA mechanisms ensure that vaccine supplies can be identified and redirected if individual officials abuse their positions and fail to distribute vaccines according to published standards or distribution plans [40].

### Findings from in-depth interviews

#### Socio-demographic characteristics of participants

Participants were drawn from four different categories of vaccine distribution stakeholders from both surveyed states: state officials, LGA officials, health facility stakeholders, and patient communities. A total of 40 IDIs were conducted across these categories: state (10; M = 5, F = 5), LGA (11; M = 5, F = 6), health facility (8; M = 3, F = 5), and community (11; M = 7, F = 4). All participants played vital roles in either the distribution or administration of COVID-19 vaccines, or were patients who shared their experiences of accessing vaccines distributed by public officials. Table 2 shows details of respondents.

**Table 2:** Demographic characteristics of respondents

Category	State A	State B	Male	Female
State-level officers	6	4	5	5
LGA-level officials	6	5	5	6
Facility-level actors	4	4	3	5
Community	6	5	7	4

#### Coordination and distribution of COVID-19 vaccines

The results show that the National Primary Health Care Development Agency (NPHCDA), which receives vaccine supplies from the federal government or donor agencies and then distributes those vaccines to states through the State Primary Health Care Development Agency (SPHCDA), coordinates COVID-19 vaccination. The State Cold Chain Officer then develops a state strategy to distribute the vaccines across all LGAs in the state through either an LGA Cold Chain Officer or a third party. At the LGA level, the LGA Immunization Officer and Cold Chain Officer distribute vaccines to vaccination teams at the ward level through a ward focal person (usually a primary health care manager).

Primary health care managers employ both fixed (e.g., health centre) and mobile (e.g., malls, markets, town halls) posts as strategic public vaccination locations, with the aim of vaccinating large numbers of people at a single location. Vaccination teams are equally dispatched to remote communities to mobilize and vaccinate consenting individuals.

To ensure accountability, vaccinators are expected to account for the number of vaccines distributed each day, which should correspond with the number of vaccination cards distributed and the number of vaccinations uploaded in the electronic data records. At the LGA level, each ward focal person is expected to return empty COVID-19 vaccine vials alongside updated vaccination records to account for the number of vaccines supplied and people vaccinated. These records must match before new vaccines are released to the focal person. Records are then sent to the state-level electronic platform before being forwarded to the national level.

#### Corrupt practices in COVID-19 vaccine deployment

##### Nepotism in hiring of COVID-19 vaccine staff

Three important components of planning for vaccine delivery were (a) recruitment of personnel, (b) contracting of personnel, and (c) budgeting. We found corruption issues across all three components.

Evidence related to the recruitment of personnel, including vaccinators, recorders, and administrative staff, shows that advertisements were not used and assessments of recruited staff were not conducted. Staff involved in the emergency deployment of COVID-19 vaccine were largely recruited informally. Those recruited were expected to show unquestionable loyalty to their recruiters and not challenge any perceived anomalies observed on the job. One informant stated:

*“The recruitment process is dishonest. People just brought in those they knew because they know that money is involved. No adverts, no screening, just people bringing relatives and friends. I am not surprised [that] accountability is poor, because you cannot challenge [those above you] when you know that you were recruited just anyhow.”* (IDI, Monitoring and Evaluation (M&E) Officer, State A)

Clear personnel contracting was also absent in the planning stage. Those recruited did not formally sign contracts, suggesting that they had no idea how long they were expected to work or how much they should expect as remuneration. Some of the personnel interviewed corroborated this lack of job transparency and highlighted that payments were arbitrary and shrouded by many opaque processes:

*“The process should be more transparent, especially when it comes to payment... They [wouldn’t] tell us [that] the job [would last] ‘from this time to that time.’ We just keep working. When we started the work, we worked March, April, ʼtil sometime around August, which was when they paid us. So, we were confused. Because we did not get a reasonable amount, and we started asking if [our pay] was monthly or daily or weekly. The mathematics was not adding up. So, we don’t know how they [paid us].”* (IDI, Vaccinator 1, State A)

When key officers were asked if they were aware of the budget plan needed for administering COVID-19 vaccines or if they were involved in formulating the budget, they said they did not know if there was a budget and that no meeting was called to inform them of how much money was received or how the received money should be used:

*“I don’t how the money is being shared, and I don’t know if issues like supervision and steady payments were incorporated into the budget. We have never contributed to the budget, and no one will show us, because if they do, they know we will raise concerns and become more watchful.”* (IDI, Assistant State Cold Chain Officer, State A)

##### Falsification and manipulation of vaccination data

Vaccinators were reportedly paid based on the number of people they vaccinated, with higher pay for higher numbers of vaccinations given. Both the government and international partners involved in vaccination efforts employed this strategy. Government and non-governmental agencies frequently sought vaccination progress data, with one participant stating *“there was this pressure everywhere for data you know, from agencies*” [IDI, Assistant Mobilization Officer, State B]. However, due to vaccine hesitancy among the patient population, vaccinators sometimes resorted to falsifying identities to increase the recorded number of vaccinated individuals.

*“[I]n giving us… a specified number [of vaccinations] to meet [per] day, I think it is bringing in forgery. I have to be frank. People don’t willingly want to be vaccinated, unless they are pressured or they have something to do with the [vaccination] card. So, in this kind of situation, how do you expect us to come up with … 20 or more [vaccinations] per day? We will definitely forge the data, especially when we know our money is dependent on the number of persons we report to have been vaccinated.”*(IDI, Vaccinator 2, State B)

Interview respondents narrated the forgery process: Vaccinators input fake identities with fake phone numbers, or they would solicit the identities and phone numbers of people they would convince to receive vaccination cards without being vaccinated. Different strategies would apply in different locations and with different people, often determined by education level.

*“In the rural areas, they go house to house to collect people’s data. They will just tell them, ‘Just give us your name and phone number that is all we just need. And they will register you.’ It is a village, so you will hardly have people to question you. But in town, you can’t just walk up to someone and tell him to give you his or her data. So, when they even accept to come for the [vaccination] cards, we are happy, and we just give [the cards] to them.”* (IDI, Vaccinator 1, State B)

The national dashboard on COVID-19 vaccination rates showed the vaccination performance of each state, and high-ranking health officials wanted to see their states perform better. During daily review meetings at the SPHCDA, which frontline vaccinators and their managers attended, there appeared to be implicit pressure on vaccination teams to ensure that sufficient numbers of the population were vaccinated. One officer involved in mobilizing communities in State B described the following:

*“Our ES [executive secretary] mandated us that we have to work, to the extent that our state will be the first or leading state in terms of the number of people vaccinated, so we devised strategies… at [one] point we were working like mad, the ES gave prizes for the best performing LGAs. We were competing, though it was a peaceful competition.*” (IDI, State Mobilization Officer, State B)

Our results indicate that “data-hungry” state officials became aware of the vaccination rate falsifications, but were reluctant to follow up with verifications and sanctions. Instead, they collated the figures and presented them as official state data.

*“It is sad that it could be that the vaccination was said to be done in State X, [but] when you call the person [recorded] the name does not match and the person will say ‘I live in State Y and I have never been to State X.’ But we do not make such calls all the time. We just take the data and submit. We know that some of them could be falsified.”* (IDI, M&E Officer, State B)

Lastly, some of the vaccinators withheld data or provided incomplete datasets because state and national agencies did not provide logistics such as transportation and internet data, and payments were delayed. This, in turn, contributed to a distorted vaccination rate picture at both the local and national levels.

*“There is a need to always provide [vaccinator] stipends and as well as subscribe… their data bundle. Another thing is to compensate them for their transportation, but all these are not regularly provided. Sometimes, it takes more than the necessary time for this money to get to them, and when it is like that, many of them will become reluctant to go to the field. When it is like that, they [are more likely to] manipulate the data or not even upload their data and synchronize them. When it is so, our data will not even speak for us at the national level. You will see us stalling behind other states. So that is the problem that we have.”* (IDI, M&E Officer, State A)

##### Nepotism, favouritism, and bribery

Some respondents reported favouring their friends and acquaintances during the vaccination process, particularly when demand for vaccines was high and wait times were long. One Electronic Management of Immunization Data (EMID) Officer in a vaccination team explicitly mentioned that sometimes they were biased when conducting services related to their position and decided to give their friends and family faster access to COVID-19 vaccines.

*“In the field, we might have about 200 people to vaccinate. We [then] queue them and start registering them. [If I] see my brother at the back, I will signal [to him], register him, and vaccinate him first. It’s corruption, but we do it anyway.”* (IDI, E-recorder, State A)

Respondents also reported payoffs. For example, sometimes service users who wanted to jump queues to gain quicker access to a COVID-19 vaccine would pay a member of the vaccination team for priority access. A service user who had been vaccinated confirmed that vaccination teams would favour people who gave payments, and sometimes users gave tips to vaccination teams.

*“The only thing that I also noticed is that people [who came after me] were vaccinated before me. That’s Nigeria for you: ‘who you know’ syndrome. Once you know someone, they will just mingle you in, they will attend to you, then you leave. That’s what they do. Some [patients] do give tips to the health workers, [and the health workers then] attend to them quickly.”* (IDI, Service user, State A)

##### Fee for COVID-19 vaccination cards

By far, the most-reported irregularities were the issuing of COVID-19 vaccination cards to non-vaccinated individuals and the collection of unofficial fees by health care workers for the distribution of legitimate vaccination cards. When COVID-19 vaccines became a strict requirement for some activities such as international travel, demand for vaccination cards increased. In this context, some health care workers issued COVID-19 vaccination cards to people who were not vaccinated but needed the document. When this happened, some members of the vaccination team demanded bribes to register these unvaccinated individuals on the online vaccination registration platform and issue them vaccination cards without actually vaccinating them. The below quotes describe this situation in more detail:

*“We cannot say somebody can sell the vaccine, but someone can sell the card. Like in those days when they say that you must have the card before travelling, someone may try to do something to give people the card without vaccinating.”* (IDI, M&E Officer, State A)

“*Some people would walk in and call you [to] the side to say that they want the card for travel, but don’t want the vaccine. No matter how hard you try to convince them, they will say no, you can see they are suspicious or afraid. They know that one can get the card without the vaccine, so they just want the card. So you help them get the card and collect small money.*” (IDI, Vaccinator, State B)

##### Demand for informal payment

In addition to collecting informal payments for vaccination cards, frontline COVID-19 vaccine officials were reported to have demanded unofficial fees for vaccinations, claiming that these fees covered logistics expenditures, internet subscriptions, and transportation. Since vaccine officials entered vaccination records on the official e-recorder platform and these e-records were used to verify vaccination certificates at border crossings, those requiring vaccination cards to engage in international travel faced heightened pressure to comply when faced with demands to make informal payments.

Higher informal payments were demanded from individuals who wanted a COVID-19 vaccine certificate but did not want to receive the vaccine.

*“Yes, I paid N5000. [The vaccinator] said… that I will pay N2000 [to cover internet subscription]. I felt bad because I wanted to take the vaccine. I decided to pay N5000 [rather] than stressing myself, because [the vaccinator] complained that [if I didn’t pay] the vaccine [wasn’t] available at the moment and I have to come back.”* (IDI, Service user, State A)

##### Remuneration irregularities

The budgeting, recruitment, and contracting processes in COVID-19 vaccine administration in Nigeria lacked transparency, with a major consequence being irregularities in paying vaccine workers. As workers were recruited informally and on a temporary bases, they were paid arbitrarily. Some state-level officers reported that they received complaints about payment variations, delayed payments, and even no payments at all. Unfortunately, the complaints were resolved either too late or not at all. Implying the possibility of forfeiture of due payments, one vaccinator stated:

*“I don’t know, but we had the issue reported at the platform, where they paid some [vaccinators] 36,000 naira, and they paid some 19,000 naira, and they paid some 22,000 naira, and they paid some 25,000 naira. These are people doing the same job. ʼTil this moment, we cannot tell why such variations.”* (IDI, Vaccinator 2, State B)

Sometimes, non-remuneration was attributed to deliberate corrupt behaviour, with reports of account details of vaccinators being swapped or account details of people who were not workers being included in the payroll. A top manager added:

*“…[S]ome will deliberately replace people’s account numbers with the account numbers of their family members. So, people that did the work won’t get the money, while the state has actually released the money.”* (IDI, Assistant State Health Educator, State B)

Payment irregularities were reported to lead frontliners, who then demanded informal payments and bribes from patients, with the rationale that they had to self-fund their transportation costs and procure internet subscriptions:

*“If we go to the field with our money and we are lucky to have people who really need to be vaccinated, we will go ahead to vaccinate them, but they must give us some money or send call credits that we can use to purchase data to upload their details as being vaccinated. That is how we cover up. There are some people that will tell you straight away to give them some money to help them in transporting themselves.”* (IDI, Vaccinator 1, State B)

#### Efforts by local establishments to control corruption in COVID-19 vaccine deployment

Some ACTA mechanisms were reported to be implemented to ensure equitable access to COVID-19 vaccines. These included maintaining the proper documentation for COVID-19 vaccine supplies, using external consultants to distribute COVID-19 vaccines, maintaining joint supervision of COVID-19 vaccination teams, conducting daily debriefings and reports of field experiences by vaccine teams, and maintaining oversight of and investigating reported irregularities.

##### Rigorous tracking of vaccine demand and supply in manual and digital formats

Both the State Primary Health Development Agency and the State Ministry have processes in place to document state-level vaccine supply and demand. The documentation process begins at the national level, where vaccines are received, and continues throughout the supply chain to the health facilities. The State Cold Chain Officer (SCCO) sends vaccine requests to the national level. When vaccines are delivered to the state, the SCCO, with support of store keepers, checks to ensure that the quantity supplied is documented both in hard copy (registers and forms) and on the open Logistic Management Information Systems (LMIS), the designated software for vaccine data collection, processing, and reporting. One respondent noted:

*“Yes, even this tablet that you are seeing was given to me for vaccine accountability. As you receive [a vaccine], you queue it in by the State Cold Chain Officer and send it to the national [level]*. *You send [the information] to the state at the same time [as you do] to the national. This gives account of every vaccine collected.”* (IDI, Disease Control and Immunization Officer, State B)

On the supply side, the SCCO is responsible for deploying vaccines to the LGA level. The Local government Immunization Officers and LGA Cold Chain Officer (CCO) receive vaccines from the state and document these deliveries using registers and forms. Officers in charge of health facilities receive vaccines from the LGA CCO and record them using registers.

*“I know that for us at LGA level, whatever vaccine that is given to us is documented. [How] you distribute it is also documented, and ledgers and data tools are there to speak for themselves.”* (IDI, Administrative Secretary, State A)

The LGA CCO is also required to count and record vaccines during vaccine collection by the officers-in-charge (OICs) of vaccination points. OICs are required to return empty vaccine vials, which are counted and recorded.

*“As you get to the local government, [where we issue vaccines] to the OICs of any health facility, you count [the doses]. [OICs] return the empty vials to me, [and we] recount [the vials] to make sure [they] tally with the number [of doses originally] given to them*.” (IDI, Immunization Officer, SMOH, State A)

EMIDs and validators also complete documentation, both as hard copies and electronic copies, at vaccination points. The use of both electronic and manual data reporting systems was established to ensure transparency and accountability in the COVID-19 vaccine distribution. This double recording helped to keep track of the number of vaccines released into communities.

*“Yes, the council has told them that any person that is immunized must be captured in the EMID record. That’s the electronic record. Apart from being captured, the activity or data must be validated to make sure that person owns that card. So, the type of vaccine that he was vaccinated with must be there, even the batch number, and this must be authenticated with the PR code.”* (IDI, M&E Officer, State A)

Occasionally, discrepancies are spotted in the data of the vaccination teams, which triggers a review process conducted by the overseers.

##### Joint supervision of COVID-19 vaccine teams

COVID-19 vaccine coordination and administration involved different levels of supervision and unannounced monitoring of health workers. In addition to the national COVID-19 committee, states established different coordination committees that were responsible for regularly supervising COVID-19 vaccine distribution and reporting at the state, LGA, and health facility levels. The state-level committee comprised stakeholders from the state’s ministry of health and SPHCDA, while the LGA team was composed of a Primary Healthcare(PHC) coordinator, Local Cold Chain Officer (LCCO), Local government Immunization Coordinator/Officer (LIO), Monitoring and Evaluation (M&E) Officer, and the Routine Immunization Officer (RIO). The facility team comprised the ward development officer, senior EMID recorder, and the health facility managers (OICs). The coordination committee was described by one participant in the following way:

*“There is a coordination committee, which comprises the state team and partners, WHO, UNICEF, and the rest of them. They meet regularly to access the progress of the [vaccine distribution] work. We are still having a meeting today, with the WHO and other teams, to ensure that the problem of payment will soon be a thing of the past. They are also monitoring the workers, and the team, to ensure that they are in the field. That’s where the supervision comes in. There is state and local government supervision, and there is also an oversight function, to ensure that the vaccine is available and in good order. If there is an event of any breakdown at the local government and state, they quickly [intervene] to make sure that things are put in place for the work to continue.”* (IDI, State Immunization Officer, State A)

The LGA team supervised the LGAs and usually focused on the administration and recording of vaccine.

*“In the LGA, we have supervisors; they come once in a while, at times twice a week to check what is going on at the vaccination site. They supervise and ask questions about the type of vaccines that we are using, how it’s been used, and the number of clients we can vaccinate that day, type and expiration date of that particular vaccine that we are using. So they are carrying supervision of the COVID work at the national, state, and LGA levels.”* (IDI, EMID Officer, State A)

Community members have also been used as local monitoring agents during vaccination deployment. Individuals have been selected and trained to work with vaccination teams to identify any potential corrupt practices in the vaccination sites.

*“ ... Even the community members have been made to be part of this process so that if such a thing occurs, it will not take time to detect and the right action taken to sanction those involved. Yes, they are mobilized and educated on what they should do. Almost all the town criers [represent] some of their communities. So anything that happens, they have strategies to communicate with the community and the community leaders.”* (IDI, M&E Officer, SPHCDA, State A)

Some supervisors performed unannounced or disguised inspections. They confirmed that, for the period this strategy was used, vaccination personnel had some level of coordination and adhered to proper practices.

*“… [I]t seems somebody disguised himself or herself from the national [level] to visit one of the facilities, and it was discovered that a particular facility in one of the local government areas was taking payments. The Executive Secretary sent me there, and I disguised [myself] as a service user and discovered it was true. It was so hot for everyone then. Everybody started behaving well […]”* [IDI, Assistant State Health Educator, State B]

##### Oversight by management in daily review meetings

Respondents described the Executive Secretaries (ESs) of SPHCDAs in the evaluated states as keen to attain high vaccination rates within their states, particularly since these rates were regularly updated on a national dashboard. As a result, ESs placed high demands on downstream vaccination personnel and field workers and would review recorded vaccination rates.

“*The ES takes the integrity of the data seriously. One of us was seriously reprimanded and made to do extra work to explain how he had more people recorded on the e-platform than the number [of] vaccine distributed. [The ES] attends the routine meetings and is always encouraging us, citing similar challenges with vaccines in the past.”* (IDI, State Mobilization Officer, State B)

Respondents, especially frontline workers, described being subject to daily thorough checks and activity reviews, especially in the early days of the vaccine rollout. Teams were responsible for reporting their activities and providing a detailed account of their inventory and the number of people vaccinated. One respondent noted:

*“We had to put in extra work. There was a routine check every day. We [would] come back and have to report our experiences. If there are any discrepancies in the data, you must have to explain it to the supervisor, else you will be reported.*” (IDI, Vaccinator, State B)

Irregularities, especially inconsistencies between the data reported online and the physical booklet or vial count, were investigated thoroughly. The ES also admonished anyone implicated in any malpractice.

*“When this news of people selling [vaccination] cards came, the ES called us and told us clearly that he [wouldn’t] be involved if anybody gets implicated in this type of thing. And we know him…so people stayed clear…especially from my team, because they know we have a tendency to report.*” (IDI, Assistant State M&E Officer, State B)

The ES also opened additional COVID-19 vaccination points so that patients could easily access vaccinations and queues could be reduced. Another respondent noted:

“*Why will somebody pay [to skip the line]?…[T]he ES made sure that we were everywhere, in front of markets, we go to churches, in the malls, we go to banks, so why will somebody want to buy [access]? Even when we had an issue of card discrepancy, he followed [up on it], got the card, traced it to the register and I think they even provided video of the person taking his second dose…”* (IDI, State Mobilization Officer, State B)

##### Accepting support from international non-governmental organizations

The majority of key informants from the state level emphasized the importance of having support from non-governmental organizations (NGOs) in promoting accountability in vaccine deployment to different LGAs in the state. NGOs provided third parties to help with vaccine deployment and supplied specialized storage facilities like deep freezers. Implementing partners also worked with states to supervise COVID-19 vaccination processes. One respondent remarked:

*“Yes, we have support from international agencies like UNICEF and then they have also contracted out the distribution [of vaccines] to a third party.”* (IDI, ES, SPHCDA, State B)

##### Positive health worker attitudes

Importantly, not all health workers engaged in corrupt behaviour. Despite incentives to engage in corruption, many health workers were observed to carry on with their duties, despite their poor remuneration and without soliciting bribes:

*“Yes, there are people that even if you give them money or you don’t, they work. There are people that are dedicated to the work, that’s the work of the health workers.”* (IDI, LCCO, State B)

Sometimes, the persistence of these workers was linked to cultures instilled by their supervisors or lead managers:

“*The ES encouraged us and told us that the [vaccine] hesitancy is normal and was experienced in past years when new vaccines were rolled out. It was hard, but as we put in more effort, we started seeing results, and it made us happy…no matter [that] the pay was not coming as it should.*” (IDI, Vaccinator, State A)

#### Corruption risks in the deployment of COVID-19 vaccines

##### Irregular payment of stipends to COVID-19 vaccination workers

Key informants commonly talked about irregular remuneration patterns. Health workers complained of not receiving their stipends and being required to cover out-of-pocket charges for internet data, transportation, and communication to facilitate their vaccination work, which in turn decreased their motivation to continue to work. As a result, high rates of absenteeism were observed among vaccinators, particularly among non-permanent staff. Some showed up some of the time, and some stopped working altogether. Staff who remained expressed that they were subject to greater performance pressure to compensate for the absent health workers:

*“I have worked for five months now, and they haven’t paid me. Some people have stopped working, while some people continue. In most vaccination sites now, if you visit them, you will see one or two persons, and others have declined… They like to manipulate the data or not even upload their data.”* (IDI, E-recorder, State B)

Payment irregularities also meant that the quality of data coming from frontline workers was compromised, which impacted the information transmitted to the national level. Supervisory level staff reported similar challenges. Workers acknowledged frustrations of working despite their salaries not being explicitly allocated in the state-level budget, noting:

*“To go to some other communities, it’s very, very far and there is no budget line for transport. The payment is not regular… maybe you will work this week [and] at the end of this week they will pay. Even though they will say they will pay you, yet nothing is forthcoming.”* (IDI, Administrative Secretary, State B)

##### Poor internet network coverage contributed to data irregularity

Key informants cited the use of an onsite electronic reporting system as a weakness because the instability of internet access led to missing vaccination data. Respondents noted that network failures while uploading data to the national server led to discrepancies between electronic records and physical registers:

*“But the other side of it is that network may fail. So, if there is a network failure, the person that has been immunized, his/her record may not be properly documented on the electronic platform. This may lead to missing data, as the person’s data will be erased from the system. … [W]e need to make sure that there is a steady network. The people who are EMID recorders should be knowledgeable enough to do the right thing at the right time. … Also to make sure that the record remains in the phone, you are not expected to [switch] off the phone even if there is no network. If this information is not provided to the EMID recorders, that data will [be missed].”* (IDI, M&E Officer, State B)

Network coverage instability meant that workers frequently risked losing data. This may have incentivized frontline workers to input false data to compensate for lost information. It may also have masked any discrepancies caused by corrupt false entries with discrepancies caused by connectivity-related data losses.

##### COVID-19 vaccine hesitancy

The combination of vaccine hesitancy and the pressure on health workers to vaccinate more people resulted in workers recording falsified vaccination rates and disseminating false information to convince more service users to take the COVID-19 vaccine. Instead of highlighting the health benefits of the vaccine, some workers resorted to using false claims to convince people into receiving vaccinations. One respondent noted that workers told people that “*the federal government said if you don’t take your vaccine, you will not get paid”* (IDI, E-recorder, State A).

Scaring people with false adverse consequences of not taking vaccines can make them more resistant and distrustful, which in turn may fuel demand for falsified vaccination cards. Another respondent noted that stronger public education on vaccines was required to promote vaccination uptake and reduce worker incentives to spread false information:

*“To make it more accountable, I think first of all there should be more awareness [about vaccines]. I think there is not much awareness, especially in the rural areas … If you let them know that taking the vaccine is for your safety, you don’t need the health personnel to sugarcoat anything... So we need more education, and people will come out by themselves.”* (IDI, E- recorder, State B)

##### Inadequate screening of COVID-19 vaccination team

Nepotism among frontline workers impacted the quality and commitment of staff distributing COVID-19 vaccines. Many individuals who were members of vaccination teams also had other engagements, which was cited as a reason for inaccurate reporting:

*“Apart from that, when you get the vaccines, you need to also screen the people that vaccinate. You get the right people that will vaccinate the people and are capable to do the work. You need to get someone that is not occupied or engaged with other engagements or events so that the person can dedicate his/her time to the work. But we do not do all these when selecting the vaccination team.”* (IDI, M&E Officer, State A)

Economic conditions worsened during the pandemic as unemployment rates increased and earnings dropped, and people were desperate for extra income. Officials in recruitment positions sometimes brought in relatives or friends to be part of the vaccination team as a way to support them. However, this resulted in some workers having little commitment to the work, which, when combined with irregular worker remuneration, incentivized corrupt behaviour.

##### Poorly specified roles in vaccine transportation

Key informants emphasized that the vaccine rollout was negatively impacted by irregular transportation systems, highlighting the need for transportation funding and reliable systems to bring vaccinators to target populations. Respondents at both the state and LGA levels reported irregular transportation funding, with no funding or transportation available to enable LGAs to transport vaccines to vaccination sites. These logistical challenges both directly limited population access to COVID-19 vaccines and created incentives among health workers to solicit informal payments from patients to cover transportation costs.

*“Vaccine transportation has become an issue… Periodically, funds may be provided to take a vaccine from the state to the LGA level but not LGA to the facility or client. Even when the fund is provided at the state to transport to the LGA level, most times [it] is not regular. That is why we are advocating for regular transport that will shift the vaccines from state to LGA down to the clients to cover the vaccination and any other periodic or supplemental immunization so that we will be sure that vaccines are available.”* (IDI, State Immunization Officer, State A)

##### Unclear reporting channels

Some respondents were unclear about to whom they should report concerns about the vaccination process. At best, reports were made to the direct line supervisor, but some respondents were concerned that effective actions were not taken to solve any potential problems. One respondent noted:

*“Sometimes when you have issues, like the e-platform not working well, I will just report to my supervisor, he will promise that things will be fixed, but you return the next day and still get the same issue occurring… [I]t can be annoying, but you have to find a way to keep going.*” (IDI, E-recorder, State B)

The absence of effective reporting points was frustrating to frontline vaccine distributors and may have contributed to personnel engaging in improper recording practices to meet prescribed vaccination targets.

#### What could be done to improve ACTA in COVID-19 vaccine distribution

##### Timely remuneration

Frontline respondents identified that timely provision of stipends and other requirements was critical. One key informant noted:

*“The federal [government] sometimes provides our stipends, and sometimes they will leave it in the hands of the state. Sometimes [international] partners, like the WHO, UNICEF, and the rest, will opt to pay [personnel]. The regularity of that payment will determine the motivation of the workers to continue [distributing vaccines]. There is a need to always provide [workers with their] stipends and [to pay for] their data bundle.”* (IDI, M&E Officer, State A)

Uncertainty regarding who remunerates workers also needs to be properly addressed to reduce the risk of corruption. Remunerations should be made promptly to sustain the motivation of the vaccination teams and ensure that they continue to do their work.

*“Promptness in [paying workers] will enhance accountability so that every personnel that is involved in this COVID-19 vaccination will [receive] his/her due stipends at the appropriate time. [This way,] people will not have an excuse to say that he/she is not being taken care of.”* (IDI, M&E Officer, State A)

##### Proper recruitment of staff in the distribution chain

Frontline vaccine distributors recommended that greater scrutiny and selection be exercised in determining who should be recruited as vaccination team personnel to ensure commitment to the job, reduce absenteeism, and promote non-falsified data reporting. One key informant noted:

“*Some workers were involved in other things and were not giving full attention [to distributing vaccines] because they had other things [to do]… Sometimes, they reported late or [did] not show up at all.*” (IDI, E-recorder, State B)

##### Strengthening of supportive supervision at all levels

National and state-level actors should provide funds for supportive supervision that covers costs for logistics and communication. Officials at the state level should encourage proper disbursement of funds for supportive supervision. Open channels of communication are encouraged to ensure adequate, timely information is properly disseminated to workers and the general public.

*“If there is a platform for ad hoc staff working to communicate to the national [level actors], it will be helpful. Staff communicates wrong information on payment of workers, and the national team always commend the state for paying all workers as written on the national platform, but we haven’t seen any money. So, if the nation makes it transparent by adding one person from each LGA, so that person can say this is what is happening, we didn’t see any money, this is what is happening.”* (IDI, E-recorder, State A)

A key informant also recommended the need for the community to provide and fund independent monitors or observers.

*“What I will say is that, during the planning, the community should be involved and participate. If they can even fund some people, like two or three persons in the community to make sure that they are independent when monitoring the vaccination team. So apart from the monitoring team, we should have independent monitors that are trained for this work. They will do independent observation and report on what is happening on this issue. So, the community members should be also involved in this independent monitoring so that they can give their independent observations and report that can be compared with others. So that will be helpful and make the process better.”* (IDI, M&E Officer, State A)

## Discussion

The scoping literature review demonstrates that inefficiencies in COVID-19 vaccine procurement and distribution are largely caused by a crisis of opacity and lack of transparency. Competition among high-income nations to secure vaccine supplies for their citizens worsened the dynamics of the vaccine supply chain and heightened uncertainty, with this pressure contributing to corruption and accountability problems. While COVAX aimed to secure supplies for countries that did not have the bargaining power to independently secure bilateral supply deals with manufacturers, it did not take substantial steps to improve transparency and accountability in the global COVID-19 vaccine procurement system.

Corruption within non-health government functions may compound the effects of corruption within the vaccine procurement system. Since effective vaccine distribution and uptake can largely depend on the trust that local citizens have in their governments, government responses to other emergencies related to the COVID-19 pandemic, such as food, security, and economic crises, further undermined public trust in government COVID-19 vaccination policies. The outcome was the emergence of corrupt activities, such as patients acquiring fake vaccine cards to navigate international travel restrictions or frontline health workers collecting exorbitant informal payments before issuing vaccine cards. These findings imply that investment in transparency should be broad and take a whole-of-country and whole-of-system approach to improve accountability and reduce opportunities for corruption.

Our qualitative study highlighted how state- and local-level actors experienced corruption in COVID-19 vaccine distribution. These informants cited examples of nepotism, favouritism, and bribery, the procurement of falsified vaccination cards, data manipulation and falsification, and the solicitation of informal payments by health care workers to conduct their normal job functions. Earlier reports [2] warning of corruption risks in vaccine procurement systems identified some of the corrupt practices that participants described in this study, such as the use of bribery to jump vaccination queues [41]. However, most of the corruption that participants described was not specifically predicted by previous COVID-19 vaccine corruption risk assessment reports.

Interestingly, participants did not describe a “vaccine rush” as forecast in several corruption risk assessment reports, which projected that corrupt actors would divert vaccines to engage in vaccine theft or solicit large informal payments for access to vaccines. Instead, an absence of a rush for COVID-19 vaccines seemed to have arisen from substantial vaccine hesitancy due to poor vaccine literacy, misinformation, and a lack of trust in the healthcare system. We found that as more people became convinced to receive the vaccine, they started coming to vaccination sites. At worst, long queues to obtain vaccines, not vaccine scarcity, was the chief concern that created an opportunity for corruption in the form of bribery to jump queues and nepotism that favoured acquaintances of health workers. Having several vaccination sites helped to reduce the long queues and opportunities for corruption.

We observed that staff recruitment in the COVID-19 vaccine distribution chain was a notable point of entry for corruption. Nepotism influenced who was recruited, and the remuneration process was poorly managed. It was not clear whose responsibility it was—state or federal government—to fund vaccine distribution logistics, and workers were left to bear the cost of transporting themselves to work and purchasing internet subscriptions to upload electronic vaccination data. Disgruntled workers, some of whom were not sure when they would be paid, faced pressure to meet vaccination targets and found ways to make money from this process. Thus, some health workers improperly solicited vaccine recipients for illicit payments to cover the costs of transportation and internet services.

Our findings show that two underlying issues contributed to this form of corruption among COVID-19 vaccine workers. First, no clear chain of command delineated who was responsible for covering the costs associated with the logistics of the vaccine distribution system. Second, falsification of workers’ human resource records was reported, resulting in the improper entry and recording of workers’ payment details. These two challenges could have been avoided with adequate planning and formalized staff recruitment programmes. More in-depth investigation is required to uncover where the systems failed, perhaps by gaining more information from national-level actors.

Our finding that COVID-19 vaccinators collected bribes to issue vaccination cards was corroborated in an earlier investigation by Media Advocacy West Africa Foundation (MAWA-Foundation). We also learned that demand for vaccine cards largely stemmed from prospective international travellers, as well as from rumours that everyone would soon require cards to receive wages and participate in government-sponsored engagements. Two lessons can be drawn from this finding for similar future health emergencies. First, as there was little impediment to accessing COVID-19 vaccines, at least in the context studied, vaccine hesitancy seemed to be a chief reason people avoided receiving vaccines but actively sought vaccination cards. Second, pressuring health workers to meet daily vaccination targets was counterproductive, resulting in health workers intentionally falsifying data to achieve the required targets. When the vaccine first became available, conspiracy theories were very popular and spread rapidly within communities and on social media platforms. Perceptions that vaccinations had been made mandatory raised fears among vaccine doubters, hardened their resolve to resist vaccines, and incentivized patient efforts to receive fraudulent vaccination cards. Resistance to receiving vaccines, and the pressure on health workers to get more people vaccinated contributed to distortions in the data on vaccination rates and other related health indices, and in turn, likely impacted both long- and short-term emergency response plans. Further, we further found evidence that when people were provided with consistent education about vaccines, they began to accept the need to be vaccinated and sought falsified vaccination cards at a lower rate.

## Conclusions

In Nigeria, local agencies adopted several measures to strengthen COVID-19 vaccine distribution accountability, including daily review meetings, frequent oversight evaluations and supervision, the use of both manual and digital records, and the involvement of third parties to facilitate distribution. We could infer from the reports of the study participants that these identified ACTA mechanisms seemed effective at promoting equitable access to COVID-19 vaccines. An adequate in-depth evaluation of these measures, though beyond the scope of the current study, would add to these findings. However, despite these measures, chronic health care worker remuneration problems appeared to compromise the entire vaccine distribution system. Correspondingly, successful vaccination programmes should ensure that staff recruitment and welfare are not compromised when deploying this sort of health emergency response. Our findings suggest that opportunities for corruption in Nigeria’s COVID-19 vaccine distribution processes were created by systemic challenges in health care worker recruitment processes, as well as poor remuneration of workers involved in vaccine distribution to patients. Although efforts were made to check for opportunities for corruption, remuneration and recruitment problems made room for corruption issues to arise.

## Data Availability

The datasets used and analysed during the current study are available from the corresponding author on reasonable request.

## Appendix

See Table 3

**Table 3:** ACTA factors that impact access to COVID-19 vaccines

Factors that may have impacted access to COVID-19 vaccines		Factors that helped or hindered ACTA in COVID-19 vaccine procurement		Anti-corruption mechanisms
International	National (Nigeria)	Help	Hinder	
Limited testing capacity led to underestimation and underpreparation for procurement needs[24]	Vaccine card racketeering: Due to lack of trust in COVID-19 vaccines, people avoided vaccines but paid bribes to get vaccination cards [28] No evidence was available to prosecute people who were reported to collect bribes for vaccine cards [28]	Shared financing and improved regional capacities for manufacturing, regulation, and procurement of tools allowed equitable and effective access to vaccines and other medical products [27]	Information around funds, disbursements, and contracts was lacking [31, 32] Financial incentives for lack of distribution logistics slowed distribution of vaccines [24] Opaque contracts were used when officials from low-income countries purchased vaccines [10, 34] Existing efficient procurement and distribution protocols were abandoned to set up new special procurement and distribution committees [2]	Prioritizing at-risk (e.g., the aged, communities with high prevalence rates) and critical groups (e.g., health workers) and proper management of the process ensures that the right people get the vaccine
High demand for vaccine but low supply may have encouraged theft and diversion [2]; Health services, logistics and distribution systems were already weak [10]	Access to information regarding how funds are received and distributed was lacking [31]	Creation of an International Pandemic Financing Facility raised additional reliable financing [24]	Exploitative vaccine manufacturers may use their product advantage during emergencies to exploit weaker countries in their aggressive pursuit for profit [47])	Global partners foresee tactical challenges and work to avert them. For example, UNICEF pushed to secure 65,000 solar cold-chain fridges for lower-income countries[43]
Existing design of vaccine distribution systems are focused on child vaccination, not massive roll outs [43] Equipment required for COVID-19 vaccine storage may differ from existing infrastructure [43]	Weak health leadership meant less readiness and poor vaccine decision-making [27] Procurement processes were hastened and procedures were waived [25]	Strengthening oversight and monitoring functions by involving local activists, anti-corruption agencies, and NGOs helped in tracking and monitoring progress with vaccines [2, 20, 36, 37](	Fake vaccines may emerge in countries with weak regulatory systems [43] Vaccines were politicized/weaponized (e.g., Russian Sputnik V vaccine was critiqued for hasty processes in getting through the scientific testing and approval process without having traceable anyway to verify if the right things were done) [42]	Oversight functions were strengthened by involving critical stakeholders, such as country activists, NGOs, and anti-corruption agencies[20, 37] National frameworks and Technical Working Groups for procurement and distribution were developed [10]
Different country systems (private vs public) may affect vaccine distribution	Arbitrary spending of other health and non-health COVID-19 resources impacted trust on government COVID policies [25]	Deploying digital platforms allowed effective monitoring of procurement and distribution processes [32]	The complexity of COVAX governance structures allows more powerful or wealthier countries and manufacturing companies to have their way and dictate who gets vaccines and when[17, 33, 34]	Efficient procurement and distribution guidelines and operating procedures that functioned pre-pandemic were maintained [32]
Vaccine manufacturers and actors interested in accountability and transparency did not have access to the same information which meant that deeper assessment of the issues cannot be made[18]				Stakeholders insist on technology transfer and waiving of intellectual property rights of vaccine manufacturer during pandemics [20, 35] Blockchain technology can be used to enhance the procurement and distribution processes [43]
Inadequate communication between government/health parastatals and the general public could trigger stigma and other hesitancy that impact vaccine adoption [44, 45]				Government and other concerned stakeholders should insist on transparency and publicizing information about vaccine contracts and specific actors [32, 46]

## Abbreviations

ACTA: Anti-corruption, transparency, and accountability
EMID: Electronic Management of Immunization Data (officer)
LCCO: Local Cold Chain Officer
LIO: Local government Immunization Coordinator
LGA: Local Government Area
LMIS: Logistic Management Information Systems
M&E: Monitoring & Evaluation Officer
NGO: Non-Governmental Organization
SCCO: State Cold Chain Officer
SMOH: State Ministry of Health
SPHCDA: State Primary Health Care Development Agency, Primary Health Care Coordinator
UNICEF: United Nations International Children’s Emergency Fund
UNODC: United Nations Office on Drugs and Crime
